# The Ministry of Health Bill

**Published:** 1921-04-02

**Authors:** 


### The Ministry of Health Bill.

Many of those who were foremost in pushing the Ministry of Health (Miscellaneous Provisions) Bill to its doom are beginning to realise, on a closer study of that badly framed but important measure of reform, that the wholesale destruction was the outcome of ignorance, and that it is essential that in one shape or another a large number of its provisions shall in the public interest again be presented to Parliament. It is even being discovered in unexpected quarters that it actually contains proposals that would have effected several large-scale economies. The County Councils Association, while getting in the usual dig in the ribs at the expense of the Ministry, that is to say, "without in any way desiring to convey approval of hasty legislation or to minimise the very proper objections taken on that ground to the Bill as a whole," makes grudgingly enough a number of admissions, "in the interests of fairness," in a somewhat patronising letter which its Council addresses to the Department.

Here are some of the acknowledged instances of "provisions of much importance in the interests of economical local administration." Clause 6 would enable local authorities to secure on advances and expenses made and recoverable by them under certain existing Acts a rate of interest corresponding with the market rate of the present day, whereas they are now able to secure such rate of interest only as was usual in pre-war times. "This would involve a direct saving to the rates of any such authority." Clause 7, empowering a local authority to supply water beyond its own district or an adjoining one, was intended and adapted to prevent the far greater expense of launching new water undertakings. Clause 11 enables existing officials to be made use of as inspectors of food instead of necessitating the appointment of new officials, a provision "both economical and, it would appear, anti-bureaucratic." Clause 14 enables sanitary authorities to be saved the expense of providing separate buildings for *post-mortem* examinations. Clause 15, sub-clause (3), would enable local authorities to use unapplied balances of loans raised before the war at a low rate of interest (which, as the law now stands, would have to be applied to the reduction of debt) for purposes which otherwise would involve raising or borrowing new money at the far higher rate of interest now current. Clause 19 enables local authorities to lend premises, if they choose, to Government Departments, and so to obviate the totally unnecessary hiring or other provision of premises by Government Departments or local authorities.

These are but a few of the host of subsidiary provisions of great practical value in the discarded Bill. That it will have to be reintroduced either as one or more measures is obvious. We sincerely hope, in the process of mutilation which it will have to suffer, whether as one big Bill or as many little ones, in order to meet the numerous criticisms, just and unjust, lavished upon it, that these useful if unspectacular provisions will be saved from the rubbish-heap.

